# Driving style recognition method using braking characteristics based on hidden Markov model

**DOI:** 10.1371/journal.pone.0182419

**Published:** 2017-08-24

**Authors:** Chao Deng, Chaozhong Wu, Nengchao Lyu, Zhen Huang

**Affiliations:** 1 Intelligent Transportation Systems Research Center, Wuhan University of Technology, Wuhan, Hubei, China; 2 Engineering Research Center for Transportation Safety, Wuhan University of Technology, Wuhan, Hubei, China; 3 National Engineering Research Center for Water Transport Safety, Institution Name, Wuhan University of Technology, Wuhan, Hubei, China; 4 School of Automation, Institution Name, Wuhan University of Technology, Wuhan, Hubei, China; Beihang University, CHINA

## Abstract

Since the advantage of hidden Markov model in dealing with time series data and for the sake of identifying driving style, three driving style (aggressive, moderate and mild) are modeled reasonably through hidden Markov model based on driver braking characteristics to achieve efficient driving style. Firstly, braking impulse and the maximum braking unit area of vacuum booster within a certain time are collected from braking operation, and then general braking and emergency braking characteristics are extracted to code the braking characteristics. Secondly, the braking behavior observation sequence is used to describe the initial parameters of hidden Markov model, and the generation of the hidden Markov model for differentiating and an observation sequence which is trained and judged by the driving style is introduced. Thirdly, the maximum likelihood logarithm could be implied from the observable parameters. The recognition accuracy of algorithm is verified through experiments and two common pattern recognition algorithms. The results showed that the driving style discrimination based on hidden Markov model algorithm could realize effective discriminant of driving style.

## Introduction

Due to terrible traffic accidents in recent years, a new approach shared by those conducting traffic safety researches has been to explore the cause of the accident and the formation mechanism from the perspective of the driver’s driving style. This approach is considered as a popular new area of research in traffic safety and traffic psychology. the driver perceived the traffic conditions, such as the changing roads, vehicles and signals by visual, auditory and other sensory.The information is formed as driving decisions after analysis and judgment of brain,then the vehicle can be driven safely by changing the runtime and direction, this series of physiological–psychological activity was called driving behavior.Driving style is the driver’s habit on driving, drivers’ driving style is the important factor when they make driving decisions, therefore, driving style is the important human factor in transportation research. Drivers behave differently in operations on gas and brake pedals due to their personalities, to show different driving style, which may be roughly classified into aggressive, moderate and mild [1, 2]. Driving style is closely related with driving behavior, especially emergency braking without warning and other dangerous driving behavior.Driving style is gradually becoming a focus of research on the evaluation of driving competence, the identification of accident drivers, the prediction of dangerous driving behavior and the improvement of vehicle braking performance.

Most research on driving style recognition commonly used a method named Subjective Global Assessment (SGA) in the past, drivers’ driving style was determined by their reports. The main means of research in the past were to apply traditional probability theory and mathematical statistics, combined with psychology to carry out subjective evaluation in the study of the relationship between driving style and driving behavior.This method is reliable but with low intelligent degree, artificial analysis needs a lot of time and energy, and it is easy to make mistakes. Findings [3] suggest that the causal effect of response time on incident clearance time will be overestimated if the self-selection bias is not considered.

## Related work

It is a new method to use the advanced artificial intelligence, information fusion and pattern recognition in the field of intelligent transportation.With the popularity of monitoring equipment, the identification of driving behavior presents as an increasingly demand of intelligent. A rich driving operation and vehicle state data are provided by the actual driving process, and these dates are increasingly collected by On–Board Diagnostic (OBD) and additional Advanced Driver Assistance Systems (ADAS), which provide technical support for the dynamic identification of driving style. Therefore, Driving style recognition model is built with the help of vehicle output data and some advanced methods, such as artificial intelligence, information fusion and pattern recognition, so it is easy to be popularized and applied with high efficiency, such as: there is a great prospect in pattern recognition with artificial neural network (ANN), support vector machine (SVM) and hidden Markov model (HMM) [4]. As recently surveyed, driver modeling remains an important issue for intelligent transportation and driving style recognition [5, 6].

Artificial neural network is a classic artificial intelligence algorithm, which analyzes the human brain’s neural network from the angle of information processing. Then a simple model is set up and a network is composed according to different ways of connection.Xu, et al. [1] used the neural network based on learning control paradigm and the real world Vehicle Test Data (VTD) to make possible the style-oriented driver modeling.Macadam, et al. [7] used neural network techniques to analyze headway data collection from a group of drivers. Pattern recognition methods were used to identify different types of headway-keeping behavior and their relative distributions. Possibilities for using neural networks to represent longitudinal control behavior of drivers were also considered and discussed.Josip, et al. [8] proposed and tested a simple but promising approach to car driving style estimation. It is envisaged to be a part of a larger warning (prevention) system.

Support vector machine was proposed by Corte et al. [9] in 1995, and it had noteworthy advantages as a small sample, nonlinear with high dimensional pattern recognition. It can be applied in pattern recognition, machine learning and artificial intelligence research.Support vector machine was applied to many areas, such as pattern recognition, regression, equalization [10, 11]. It was adopted in applications such as dynamic robot control [12], space robot control [13], image classification [14], human dynamic gait recognition [15]. Nonetheless, the support vector machine is more sensitive for missing data; there is no universal solution for a nonlinear problem, so kernel function must be chosen carefully to deal with it.However, support vector machine is usually used for a binary solution but it is short of time series.

As for the strong time sequence of driving behavior, the above two methods are not good for the interpretation of driving behavior. There is an innate advantage of hidden Markov model in dealing with time series data, such as its good learning ability of network structure and good prediction efficiency of the process; hidden Markov model is often used to predict the perilous state of the production site. Using the proposed machine learning method hidden Markov model, the individual driving behavior model is derived and then the procedure is demonstrated for recognizing different drivers through analyzing the corresponding models. Meng, et al. [16] defined performance measures for evaluating their resultant learning models using a hidden Markov model based similarity measure, which could be helpful to derive the similarity of individual behavior and corresponding model. At present, there is little field that hidden Markov model is used to research driving style. Driving operation and driving condition can satisfy the double random process of hidden Markov model, which can be used to establish the relationship between driving behavior and vehicle motion for a set of driving behavior observed sequence [17].

Hidden Markov model usually consists of Markov chain and general stochastic process, the former is used to describe the transfer of the state, the latter is used to describe the relationship between the state and the observation sequence, each driving condition is corresponding to an observation sequence (i.e., driving style), there is no contact of these observation sequence on the surface, but they are corresponding to the hidden states of a Markov chain, because the current driving condition is usually related to its state a moment ago only, though this state cannot be measured directly, but it can be used to describe the changes of driving condition by hidden Markov model.

In this paper, it aims to the driver’s driving characteristics in China, the real vehicle experiment road was carried out, the driver’s driving style was recognized by hidden Markov model, the drivers’ subjective evaluation was used to verify identification. In addition, a more efficient rate of driving style can be got compared with artificial neural network and support vector machine. First of all, braking impulse and the maximum braking unit area of the vacuum booster within a certain time are collected in braking, general braking and emergency braking, distinguished and coded. The aggressive, moderate, mild driving style is trained to build hidden Markov model based on driver braking characteristics. Finally, driving style recognition is realized based on hidden Markov model with a different degree of emergency braking characteristics as input. According to the comparison with the neural network and support vector machine, the recognition runtime and accuracy of the approach proposed in this paper are improved.

## Experiment procedure

The driving style was derived from long term driving habits, the goals were to test drivers with rich and longer driving experience. The sequence of driving style and braking behavior constitute a Markov chain. We collect the human driving data directly for HMM training, which includes braking only. We do not utilize other car dynamics and environmental variables as the inputs, such as the car’s yaw angle with respect to the road, lateral offset to the road’s center, the road curvature, etc, which benefits the efficiency and robustness of the system.

Testing scenes mainly involved the city expressway. The driving scene consisted of prepared roads and testing roads. Prepared roads were 2km long, which was the vehicle’s start of every test. The main purpose was used for the vehicle’s acceleration; namely, accelerating from standstill to the desired speed. The traffic flow was free. In order to eliminate the influence of the road alignment, the research section of a city expressway which the change of the subjects vehicle’s yaw rate was very little intercepted by sensors. In order to eliminate the driver’s individual differences, the individual factors (gender, age, driving experience, level of education, etc.) of the subjects should obey the uniform distribution as far as possible.

Before experiment, all subjects gave their informed consent for inclusion before they participated in the study. The study was conducted in accordance with the Declaration of Helsinki, and the protocol was approved by the Academic Committee of Intelligent Transportation System Research Center, Wuhan University of Technology. The formal experimental procedure was as follows.

- Before the test, subjects were asked to complete a questionnaire on their basic information: age, gender, driving experience, whether myopic or not, whether the subject underwent a brain operation, whether the subject had a cold, and whether the subject drank coffee or other stimulating drinks and drugs that would affect brain function before the test. All subjects that did not meet the conditions were removed. At the same time, subjects were required to provide their signatures for the reward.
- The staff explained the test procedures and ensured that all subjects understood the test requirements and contents before practice. Silence must be maintained in the control room and all communication tools should be turned off.
- At the end of the experiment, all participants were asked to complete driving styles inventory, in order to subjectively evaluate driving style.

### Participants

The number of subjects was 30, there were 15 professional drivers and 15 staffs and students from Wuhan University of Technology (20 male subjects and 10 female subjects). They were aged from 22 to 55 years old (Mean age = 32.2, SD = 8.2). They had driving experience from 2 to 18 years and 6.9 years’ driving experience on average, the driving distance was up to 6500 km in the experiment. It should be noted that there are many people who have driving license without actual driving experience in China. In order to avoid interfering with the credibility of the experiment, driving mileage was used instead of driving license age as an indicator of drivers’ proficiency in this research. In terms of driving mileage, they drove an average of 110,000 kilometers, ranging from 400 to 400,000 kilometers.

### Vehicle instrumentation

The road traffic experimental car was developed and integrated based on the GAC Trumpchi car in Intelligent Transport System Research Center, Wuhan University of Technology, which provided platform support for a real vehicle road test and data collection.The field test was carried out on the third ring expressway in Wuhan from September 14^*th*^ to 30^*th*^, 2016 and from March 12^*th*^ to 28^*th*^, 2017. The experimental car was equipped with a variety of data collection equipment. A road environment image acquisition system was used to record data, such as driving operation and road scene, and was also used for the calibration of emergency braking behavior(S1 Fig [18]). Driving behavior detection systems and an on-board data acquisition system are used to obtain the vehicle running information and record vehicle traffic conditions. Information such as the driving operation and vehicle movement is extracted from the collecting platform by configuring the CANoe analyzer.

### Test route

Considering the influence of different types of roads, the mixed-route in and around Wuhan (city road, city expressway, highway) were selected for experiments. The city roads section consisted of two or three lanes in each direction and the speed limits varied from 40 to 60 km/h. The total distance of urban roads was about 12 km. The urban expressways section consisted of three or four lanes in each direction and the speed limit was 80 km/h. The total distance of urban expressways was about 34 km. The freeways section consisted of three or four lanes in each direction and the speed limits varied from 100 to 120 km/h. The total distance of freeways was about 45 km. As shown in S2 Fig. The first segment consisted of a short adaption drive through an expressway which speed limits was 70 km/h indicated by yellow in the figure, in order to let drivers familiar with the vehicle condition. The second segment was the freeways which indicated by blue in the figure. The next two segments were urban expressways and urban roads indicated by red and green in the figure, respectively. Participants were given route guidance instructions provide by an experiment assistant present in the vehicle all the time in the experiment. All participants drove the same instrumented vehicle throughout the study.

## Methodology

### Subjective style evaluation

Real driving style was obtained by the subjective global assessment. The experimental driver reported their driving style after the experiment, their driving style were shown in S1 Table. In the thirty drivers’ driving style samples list, ten drivers were of aggressive driving style samples, ten were of moderate driving style samples and ten were of mild driving style samples.

### Feature extraction and coding

In order to analyze the braking characteristics, the typical braking data collected in the experiment is analyzed in this paper. After the driver received the braking signal, the relationship between the brake pedal force, braking deceleration and braking time is as shown in S3 Fig. The CAN bus was used to obtain vehicle data such as brake pedal information, and so on. All data was synchronized with the master time that was transmitted by the monitoring software at every 0.1s.

Feature extraction is one of the key points in pattern recognition. As shown in the study, the driver’s acting force of brake pedal presents as obvious priorities in the braking process. The driver exerts a greater force on the brake pedal in a short time to avoid a collision. The braking pressure changing rate is shown as an important feature, and the driver’s braking behavior is influenced by driving style [19]. This feature is considered as the focus. Considering the braking performance of vehicle, the critical value of conflict severity was obtained by the ratio of braking distance to instantaneous speed. The quantization value of conflict severity was established taking conflict time and critical value as parameters, and the severity of traffic conflict was quantified and classified [20, 21].So the typical braking data collected in the experiment was analyzed in this paper.

S3 Fig was applied to analyze the typical braking progress and show the change of braking characteristics with time directly, which contained braking impulse, maximum value of braking force and the time window. The abscissa, ordinate and corresponding symbol of this figure are shown as below. In the braking process, suppose that there was not slip phenomenon caused by lock of the wheels, when the brake pedal force *F*_*p*_ or brake fluid pressure *p* rises to a certain value (brake fluid pressure *p*_*a*_), brake force *F*_*μ*_ reaches to the ground adhesion *F*_*φ*_ and stop rising, with the invariability of brake structure and external conditions, the vacuum booster pressure *p*_*μ*_ is in direct proportion to brake force *F*_*φ*_ and brake pedal force *F*_*p*_ [22]. *p*_*μ*_ (the unit is Mpa) is up to the peak and down slightly. When there is pressure to the brakes, slippage is usually 20% 50% of the peak [23], and then a slight upward trend appears. The reason for *p*_*μ*_ is at early stage of the brake, *p*_*μ*_ is increasing with the increases of tire-road adhesion coefficient *φ*; *p*_*μ*_ is declining when there is a big difference between the adhesion coefficient and sliding adhesion coefficient. At a later stage of the brake, $F_{\varphi }^{\prime }$ is increasing with the lower runtime, which leads to a slight increase of *p*_*φ*_ [22]. The braking curve is divided into two parts; braking process within the brake work time *τ*_1_ is called as transient process, and it is called stationary process in the continuous braking time *τ*_2_. The essence of the transient process is the constantly increasing tire deformation. On the other hand, tire deformation of the smooth process remains unchanged basically. *τ*_1_ is decided by the structure of the brake, and it is also an important influence on braking distance; *τ*_2_ is the real reason for slowing the car.

All data is output force (the unit is Mpa) of vacuum booster in S3 Fig, the original data has been uploaded already. It is found that the degree of the emergency brake is related to braking impulse per unit area *p*_*i*_, and the maximum value of braking *p*_*max*_ in a certain time *T* based on a lots of experiments.

$$\begin{array}{c} p^{*}=\lim_{f\to \infty }\sum_{i=1}^{Tf}\frac{p_{i}}{f} \end{array}$$ (1)

$$\begin{array}{c} p_{max}=max\left\{p_{i}\right\},i\in \left[1,Tf\right] \end{array}$$ (2)

As shown from the experiments, *T* is the time window, it is 1.6s, *f* is the sampling frequency, the value is 10, so the Eqs 1 and 2 can be simplified as:

$$\begin{array}{c} p^{*}=0.1\sum_{i=1}^{16}p_{i} \end{array}$$ (3)

$$\begin{array}{c} p_{max}=max\left\{p_{i}\right\},i\in \left[1,16\right] \end{array}$$ (4)

As shown in real vehicle tests for many times on the third ring expressway in Wuhan, p* = 80Mpa⋅Hz, *p*_*max*_ = 9Mpa is the critical value of general braking and emergency braking. Braking behavior data was coded, with 1 representing the condition of general braking and 2 representing the condition of emergency braking. General braking requires that braking impulse was more than 80Mpa⋅Hz, and the maximum value of braking force was less than 9Mpa in the time window (1.6s, after start of braking behavior); otherwise, braking behavior that not fitted conditions of general braking was called as emergency braking. Coding rules are as follows:

$$\begin{array}{c} C=\{\begin{array}{cc} 1, & p^{*}>80\&p_{max}<9\\ 2, & otherwise \end{array} \end{array}$$ (5)

As shown in studies, the fit of data is the best when each observation sequence in eight data. Considering model simplicity, each observation sequence has eight braking data [24]. The data preprocessing is completed by MATLAB. The brake pedal pressure for starting point of sampling is bigger than 1Mpa, each duration is 1.6 s, the recorded 30 subjects (each subject have 60 braking data) with a total of 1800 times car brake data is the sample of study, such as braking time, brake information of pressure booster. The braking feature coding *C* is calculated by Eq 5.

### Driving style modeling and solution

The theoretical foundation of HMM is established by Baumet al., which is promoted by Rabiner and others, which has strong modeling ability to the time domain signal so that it has become a research hotspot [16, 25]. It has been successfully used in speech recognition, behavior recognition, and character recognition and fault diagnosis. HMM is a powerful statistical tool on describing discrete time data samples, it is a double random process, one of which is the Markov chain, which is a basic stochastic process to describe the transfer of state. Another stochastic process describes the statistical correspondence between States and observations. Because the state cannot be seen directly, HMM is a random process to perceive the existence of the state and its characteristics. A hidden Markov model can be defined by:

{*S*} –a set of state including an initial observation state *S*_*M*_ and a hidden state *S*_*N*_;
*A* –the transition probability matrix, *A* = *a*_*ij*_,where *a*_*ij*_ is the transition probability of taking the transition from state *i* to *j*;
*B* –the output probability matrix, *B* = *b*_*j*_(*O*_*k*_) for discrete hidden Markov model or *B* = *b*_*j*_(*x*) a continuous hidden Markov model, where *O*_*k*_ stands for a discrete observation symbol, and *x* stands for continuous observations of *k*–dimensional random vectors.

If the initial state distribution *π* = {*π*_*i*_}, the complete parameter set of the hidden Markov model can be expressed compactly as λ = {*N*, *M*, *A*, *B*, *π*}.

#### Model initialization

The initialization of hidden Markov model is to confirm the original value of hidden Markov model a set of five parameters λ = (*N*, *M*, *A*, *B*, *π*). The sequence of driving style and braking behavior constitute a Markov chain. The number of hidden states (driving style) is 3, which are aggressive, moderate and mild; the number of observation status (braking behavior) is 2, which are general breaking and emergency breaking. And it is necessary to consider two factors to observe the number of sequences: the recognition accuracy and the recognition duration of the algorithm. If the sequence is too long, then the matrix dimension is too high, recognition duration is reduced with amount of calculation; if the sequence is too short, it is difficult to reflect the relationship between each node of the Markov chain, which would result in lower recognition rate. According to set the sequence of different length, it can be found that when the sequence length is 8, the fitting effect of the model is the best [24]. For the observation sequence length *T*, which should be considered whether it can describe the braking characteristics sufficiently, *T* is fixed as 8 in the experiment. Eight consecutive braking characteristics constitute an observation sequence.

Traditional incident clearance time studies rely on statistical models with rigorous assumptions [26]. It is generally believed that the initial parameter selection has little effect, which can be randomly or evenly selected [27]. The initial state probability distribution vector can be selected as: *π*_0_ = (1, 0, ⋯, 0)′. the initial probability distribution initialization of matrix *A* is uniform distribution, the scale of matrix *A* is 3 × 3, *A*_0_ = [*a*_*ij*_]_3×3_, *a*_*ij*_ = 1/3. The initial probability distribution of mixed matrix *B* is confirmed by different braking characteristics prior probability. According to the selection of the number of hidden states *N* and the number of observations *M*, the scale of matrix *B* is 3 × 2, and the initial probability distribution of matrix *B* is uniform distribution, *B*_0_ = [*b*_*ij*_]_3×2_, *b*_*ij*_ = 1/3.

After stipulation of the initial value of hidden Markov model parameters, the initial model of the hidden Markov model λ_0_ can be obtained.

#### Iterative algorithm

Baum-Welch iterative revaluation algorithm is put forward to solve the problem of parameter estimation of HMM, it is an optimization algorithm based on steepest gradient descent, a derivative only need to solve the loss function calculation with small price, so the convergence speed is fast, it is one of the most reliable training algorithm of HMM [28].

According to the division of driving style, each driving style is trained with coresponding hidden Markov model. The Baum-Welch under multiple observation sequence algorithm [25] is used to carry out driving style training. Each model of the driving style is established to realize that the driver’s difference could be reflected in the driving style model, each model of the driving style is applied in the score evaluation of unknown data sequence, the corresponding models of homologous maximum Log-likelihood value is selected as the matching driving style and unknown data judgment function is realized. At the same time, log-likelihood can also explain the similarity of model driving style and judge the matching degree of features and model.

In order to judge the driving style, the problem of hidden Markov model learning and training is solved firstly to establish a basic model. At present, the processing method about hidden Markov model learning that is widely used is estimation algorithm Baum-Welch. This is an iterative algorithm, the given empirical estimates of the parameters as initial value, and the optimal value of each parameter gradually tends to be more reasonable through continuous iteration. The algorithm can be simply described as follows:

$$\begin{array}{c} \xi_{t}\left(i,j\right)=\frac{P(q_{t}=i,q_{i+1}=j,O|\lambda )}{P(O|\lambda )} \end{array}$$ (6)

$$\begin{array}{c} {\bar{a}}_{ij}=\frac{\sum_{i=1}^{T-1}\xi_{t}\left(i,j\right)}{\sum_{i=1}^{T-1}\gamma_{t}\left(i\right)} \end{array}$$ (7)

b¯jk=∑i=1Ot=VkT-1γt(j)∑i=1Tγt(j) (8)

Whereby $\gamma_{t}\left(i\right)=\sum_{j=1}^{N}\xi_{t}\left(i,j\right)$ is the probability of *S*_*i*_ on the time *t*, $\gamma_{t}\left(j\right)=\sum_{i=1}^{N}\xi_{t}\left(i,j\right)$ is the probability of *S*_*j*_ on the time *t*, $\sum_{i=1}^{T-1}\xi_{t}\left(i,j\right)$ shows the expectation of transitions numbers from *S*_*i*_ to *S*_*j*_, $\sum_{i=1}^{T-1}\gamma_{t}\left(i\right)$ shows the expectation of roll out numbers from the status *S*_*i*_ in the whole process, 1 ≤ *i* ≤ *N*, 1 ≤ *j* ≤ *N*, 1 ≤ *t* ≤ *T* − 1.

#### Recognition algorithm

The dynamic programming is used by the forward backward algorithm to transform the multi-stage process into a series of single stage problems. The relationship between the various stages is used to calculate one by one, as the low complexity, the algorithm can ensure the high accuracy of recognition and the recognition speed is improved [15]. In addition, the forward backward algorithm is a classic HMM efficient reasoning algorithm with the advantages of intuitive expression and easy programming [29], and without complex transformation.

The Forward-Backward algorithm [30] is used to identify the driving style. Under the given model λ, an observed sequence is output at the time *t*, and the probability of status *S*_*i*_ can be represented forward variable as:

$$\begin{array}{c} \alpha_{t}\left(i\right)=P(O_{1},O_{2},\cdots ,O_{t},q_{t}=S_{i}|\lambda ) \end{array}$$ (9)

Recursive equation is as follows:

$$\begin{array}{c} \alpha_{t+1}\left(j\right)=\left[\sum_{t=1}^{N}\alpha_{t}\left(i\right)a_{ij}\right]b_{j}\left(O_{t+1}\right) \end{array}$$ (10)

And, *α*_*t*_ = *π*_*i*_ *b*_*i*_(*O*_1_).

The following equation is applied to assess the probability of an observation series of corresponding state:

$$\begin{array}{c} P\left(O|\lambda \right)=\sum_{i=1}^{N}\alpha_{T}\left(i\right) \end{array}$$ (11)

#### Algorithm procedures

The aim here is to collect some braking behavior sample and form a sample set for a particular driving style category in the overall process of training. A hidden Markov model is initialized according to the driving style characteristic of the class, and the sample set is used to train. The process of identifying shows that an observation series is input respectively to three driving style hidden Markov model, the probability of three hidden Markov model driving style is obtained and compared with each other. The one with the maximum probability is the corresponding driving style of this observation series. S4 Fig shows the training and identification flow chart.

Specific training of driving style and recognition process are as follows:

- Step One: The training sample set of driving style *c*_1_ is given, which contains *T* observation sequence, *n* is for the iterations, *π*_*i*_ = *γ*_1_(*i*), the expectation in the status *S*_*i*_ when *t* = 1.
- Step Two: Start the training of the *t* observation sequence.
- Step Three: After the given observation sequence and model parameters (the first iteration is model parameters of initialization λ_0_), using Eq 11 to calculate the forward variable *P*(*O*|λ).
- Step Four: Calculate *γ*_*t*_(*i*) and *ξ*_*t*_(*i*, *j*).
- Step Five: Traversal *t*, accumulate *γ*_*t*_(*i*) and *ξ*_*t*_(*i*, *j*), hold the result and back to step two, choose the next observation sequence to calculate till *t* = *T*.
- Step Six: Using revaluation Eqs 7 and 8 to reappraise the model parameter and obtain the new model λ′.
- Step Seven: Judging the model parameter iteration convergence condition, then using the forward probability in model evaluation, comparing the two iterative calculations so that the forward probability *P*(*O*|λ) and *P*(*O*|λ′) are obtained.
- Step Eight: If |lg *P*(*O*|λ) − lg *P*(*O*|λ′)|<*ε*, then the model convergence, *ε* is the predefined threshold, which is chosen as 0.001. The best hidden Markov model of driving style *c*_1_ is obtained, then next training of driving style *c*_2_ could begin; Otherwise, a new model λ′ has taken place of the original model λ, then turn to step two when the number of iterations increases again, and the next iteration could begin. hidden Markov model λ_*aggressive*_, λ_*moderate*_, λ_*mild*_ for three driving style would be obtained after training.
- Step Nine: The corresponding model lg *P*(*O*|λ_*i*_) of maximum likelihood logarithm of unknown sample sequence is the driving style category.

## Results

### Recognition result by hidden Markov model

There is a total of 1800 times car braking data, each subject has 60 times car braking data, 48 times braking data of each subject is chosen to train, and the 48 times braking data is divided into 6 samples, a sample means to take 8 constant breaking data as a sequence, the number of samples that used for training is 80% (1440/1800) of the total samples. That is to say each observation sequence consists of 8 braking feature values, which means each sequence includes 8 brake, in the whole experiment, 30 subjects had an average of 60 braking for each one, that is 30 × 60 = 1800 brake, then the 60 braking for each person is divided into 6 non repetitive sequences with each observation sequence as a the sample, there are 30 × 6 = 180 samples. The Baum-Welch algorithm was used to train and model the vector data 30 × 6 among them. Training log-likelihood changes were shown, according to the research situation. The log-likelihood of three driving style could reach a steady state with at most 40 times iterative training; they were stable about -31, -37, and -50, respectively. The corresponding driving style type of the maximum logarithmic likelihood values were homologous to three driving style such as aggressive (S5 Fig), moderate (S6 Fig) and mild (S7 Fig).

The Forward-Backward algorithm was used to assess vector data 30 × 1 of eight braking operations for each group in the remaining 30 groups. The maximum log-likelihood was obtained by identify verification, which showed that the similarity of driving style and matching degree of judgment between characteristics and model reaches maximum. There were 8 times for wrong identification, which the aggressive driving style and mild driving style identified falsely for 4 times respectively; the remaining 26 were correctly identified.

In this paper, the method of identifying the correct rate is used to evaluate the driving style discrimination. The Eq 12 is used to calculated the accuracy of driving style recognition, the accuracy *μ* of driving style recognition shows the rate of times for correct recognition in the total times of recognition, *c*_*k*_ stands for driving style, in which *k* ∈ {*aggressive*, *moderate*, *mild*}.

$$\begin{array}{c} \mu =\frac{T_{aggressive}+T_{moderate}+T_{mild}}{S} \end{array}$$ (12)

In the Eq 12, *T*_*aggressive*_ is the times that samples are recognized as *c*_*aggressive*_ and actually are attributed to *c*_*aggressive*_, *T*_*moderate*_ is the times that samples are recognized as *c*_*moderate*_ and actually are attributed to *c*_*moderate*_, *T*_*mild*_ is the times that samples are recognized as *c*_*mild*_ and actually are attributed to *c*_*mild*_, *S* is the whole times for samples recognition.

Recognition accuracy and model recognition time were used for the evaluation model. In the 30 times tests, there were 8 times identification errors in a total, and the recognition accuracy was 88.86%. The program runtime was recognized through the MATLAB command tic/toc recognition program. It was concluded that the hidden Markov model recognition runtime was an average of 0.02s after repeated experiments.

### Recognition result by back propagation neural network

The data source of back propagation (BP) neural network is the same as the former two schemes. The structure of the BP network features is introduced as follows. Firstly, the data is modularization processed, eight input neurons is set by eight consecutive braking information which are also the characteristics of the eight dimensions; and then two output neurons are built and coded. The first kind of coding is “01”, the second kind of coding is “10” and the third kind of coding is “11”. Three output neuron represents three driving style. Due to a hidden layer which can fit various kinds of classification, the BP network is designed as a layer. The number of internal nodes in the hidden layer is namely 30 hidden layer neurons according to 2 layers the design of the number of input layer.

The vector data of 30 × 6 is taken as the training sample. The BP neural network is modeled with the help of the MATLAB neural network toolbox. A random number of weight threshold (−1, 1) generate and sigmoid function is used in the activation function.

Training was stopped when all the training samples of mean square error (MSE) and less than 0.01 or the number of iterations reached 1000 times. The gradient descent method is used to correct each sample weight, the update rate of the decision value is determined by learning factor and the learning factor is set as 0.8 after repeated experiments. The optimization method is to add momentum in this paper; the momentum factor is set as 0.9. The BP neural network is used to judge the rest of the 30 × 1 vector data. Driving style model identification accuracy is 77.27% and the recognition time is 1.25s.

### Recognition result by support vector machine

In this paper, the problem to be solved is about one class classification. Support vector machine involves two classification methods. Thus, the three categories need to be divided into three binary classification problems, which can use a directed acyclic graph (DAG) to describe the relationship of these classifiers [31]. Driving style is represented by A, B, C. At the time of extraction of the training set, a corresponding vector is extracted as set respectively and C corresponding vector is extracted as a negative set; a corresponding vector is as positive set and B corresponding vector as negative set; when B corresponding vector is the positive set, the C corresponding vector is the negative set. The three training sets are trained respectively, and three training results files are obtained, such as A (aggressive) and B (moderate), C (mild).

At present, each support vector machine classifier training is under the help of the modeling forecast simulation platform (CMSVM) that more widely used [32], three support vector machine are needed: AvsC-SVM, AvsB-SVM, the BvsC-SVM.

In order to optimize the model, all data samples need to randomly be divided into three parts: the training sample subset used for modeling, the experimental sample subset used to optimize the model parameters and sample subset used to test model generalization of the test [32]. The three sample set are input into CMSVM in sequence, and the recognition accuracy of AvsC-SVM, AvsB-SVM and BvsC-SVM are 83.32%, 81.84% and 83.33%, respectively. Recognition runtime is 0.15 s, 0.18 s and 0.17 s, so the recognition accuracy based on CMSVM is a product of three support vector machine recognition accuracy is 82.83%, and the model runtime is the sum of three support vector machine: 0.15s+max(0.18s, 0.17s) = 0.32s.

## Conclusions and discussions

Three kinds of model recognition accuracy was shown in S2 Table, and three kinds of model recognition runtime was shown in S3 Table. Viewed from recognition accuracy and runtime, the hidden Markov model is the best method, followed by support vector machine, artificial neural network is the worst.

Josip’s study [8] collected car engine data through OBDII interface, proposed approach based on neural network demonstrated the same accuracy as this paper, and mean squared error of 0.0353 on the test dataset acquired. Qian, et al. [33] discussed the application of the multi class support vector machine classifiers and compared the performance of different support vector machine parameters. Support vector machine with polynomial kernel performed better than other functions. Their results demonstrated that the SVMs have the potential to obtain a reliable distinction among the testing human subjects, individual identification can be recognized with the multi class SVM’s classifiers with a success rate of over 85%.Pentland, et al. [34] used these dynamic Markov models to recognize human behaviors from sensory data and predict human behaviors over a few seconds time, in order to test the power of this modeling approach, an experiment was reported in which 95% accuracy were able to achieve at predicting automobile drivers’ subsequent actions from their initial preparatory movements.Thus, it is obvious that the hidden Markov model recognition effect is better than the support vector machine and artificial neural network.

Vehicle braking behavior is preprocessed by the algorithm put forward in this paper. The vehicle braking data is operated as reading and sampling. Braking characteristics are coded to obtain hidden Markov model observation sequence for vehicle braking behavior; the judgment of driving style is realized by the hidden Markov model training sample driving style and pattern recognition based on it. And, real driving style is obtained by the subjective global assessment.Finally, the advantages and disadvantages of recognition accuracy and runtime for hidden Markov model, support vector machine and artificial neural network are compared. Comprehensive hidden Markov model recognition accuracy is 88.86%, and it reached 99.9% of hidden Markov model moderate driving style. The average recognition runtime is 0.02s. Comprehensive recognition accuracy of support vector machine is 82.83%. The average recognition runtime is 0.35 s. Comprehensive recognition accuracy of artificial neural network is 77.27%. The average recognition runtime is 1.25s. Experimental results show that driving style can be identified quickly and efficiently by the proposed hidden Markov model algorithm.

In this paper, an experimental system has been established that can capture and analyze driving behavior in real vehicle driving environment. The hidden Markov model (HMM) was used to train the driving style samples, and the performance of the proposed algorithm was evaluated. The results showed that the method was effective and the recognition rate was about 88.57%. Aims to the driving comfort demand, an intelligent driving assistance method is proposed, the different driving style can be identified through the capture and analysis of the driving behavior, the advantage of the proposed method is effective to extract the dynamic characteristics of the driving style. Driving style recognition can be used to set the warning threshold of different safety driving system and improve the driver’s acceptance of ADAS. As considering for more driving behavior indicators, a comprehensive driving style rules library is established to apply in the high precision driving style recognition system. At present, it is a single braking index, braking parameters and vehicle kinematics will be combined in the future. In addition, other methods are needed in terms of the improvement of HMM.

## Supporting information

S1 Fig Road traffic experimental equipment. Reprinted from “Nengchao Lyu, et al. Clustering-Based Lateral Longitudinal Target Recognition of In-Vehicle LIDAR Data. Cota International Conference of Transportation Professionals 2016 Jul:308-321.” under a CC BY license, with permission from Nengchao Lyu, original copyright 2016.
(TIF)

S2 Fig Selected experimental roads. The first segment consisted of a short adaption drive through an expressway which speed limits was 70 km/h indicated by yellow in the figure, in order to let drivers familiar with the vehicle condition. The second segment was the freeways which indicated by blue in the figure. The next two segments were urban expressways and urban roads indicated by red and green in the figure, respectively.
(TIF)

S3 FigPressure change of automotive braking vacuum booster.*p*_*μ*_ is up to the peak and down slightly. $F_{\varphi }^{\prime }$ is increasing with the lower runtime. The braking curve is divided into two parts; braking process within the brake work time *τ*_1_ is called as transient process, and it is named stationary process in the continuous braking time *τ*_2_.(TIF)

S4 Fig Flow chart of identification for driving style hidden Markov model training. The one with the maximum probability is the corresponding driving style of this observation series.
(TIF)

S5 Fig The forward scores for hidden Markov model #1 (Aggressive). The log-likelihood of aggressive driving style could reach a steady state with at most 40 times iterative training; it was stable about -31.
(TIF)

S6 Fig The forward scores for hidden Markov model #2 (Moderate). The log-likelihood of moderate driving style could reach a steady state with at most 40 times iterative training; it was stable about -37.
(TIF)

S7 Fig The forward scores for hidden Markov model #3 (Mild). The log-likelihood of mild driving style could reach a steady state with at most 40 times iterative training; it was stable about -50.
(TIF)

S1 Table Driving style of experimental subjects. Table notes subjects’ driving style.
(DOCX)

S2 TableComparison of recognition accuracy.Accuracy $\mu =\frac{T_{aggressive}+T_{moderate}+T_{mild}}{S}$.(DOCX)

S3 Table Comparison of recognition duration. Calculated by the MATLAB command tic/toc recognition program.
(DOCX)
