# Supplementary figures and images for: Driving style recognition method using braking characteristics based on hidden Markov model
Source: PLoS One. 2017 Aug 24;12(8):e0182419. doi: 10.1371/journal.pone.0182419 (PMC5570378; doi:10.1371/journal.pone.0182419)

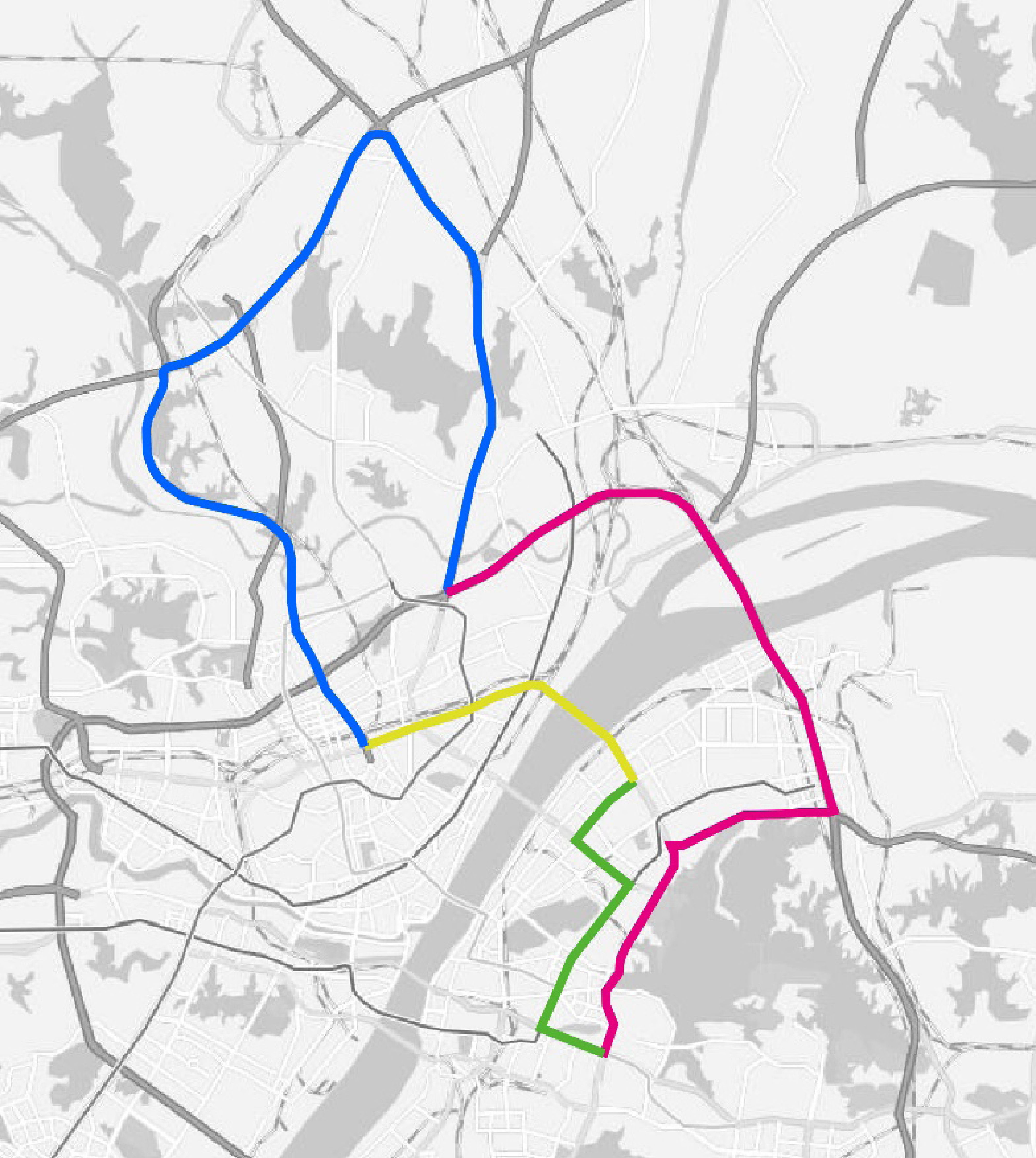

Supplement: S2 Fig — The first segment consisted of a short adaption drive through an expressway which speed limits was 70 km/h indicated by yellow in the figure, in order to let drivers familiar with the vehicle condition. The second segment was the freeways which indicated by blue in the figure. The next two segments were urban expressways and urban roads indicated by red and green in the figure, respectively. (TIF) [file pone.0182419.s002.tif]

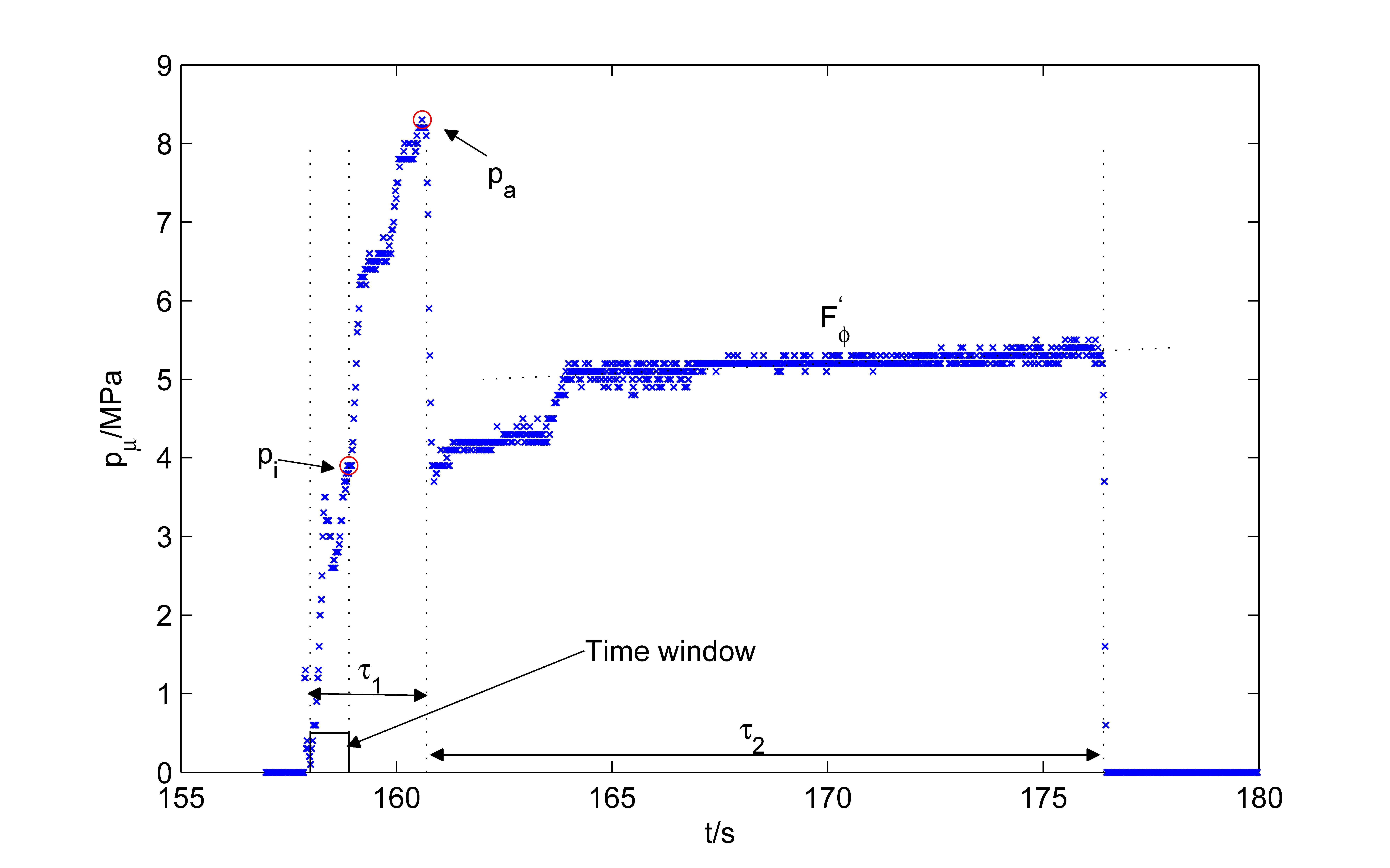

Supplement: S3 Fig — pμ is up to the peak and down slightly. Fφ′ is increasing with the lower runtime. The braking curve is divided into two parts; braking process within the brake work time τ1 is called as transient process, and it is named stationary process in the continuous braking time τ2. (TIF) [file pone.0182419.s003.tif]

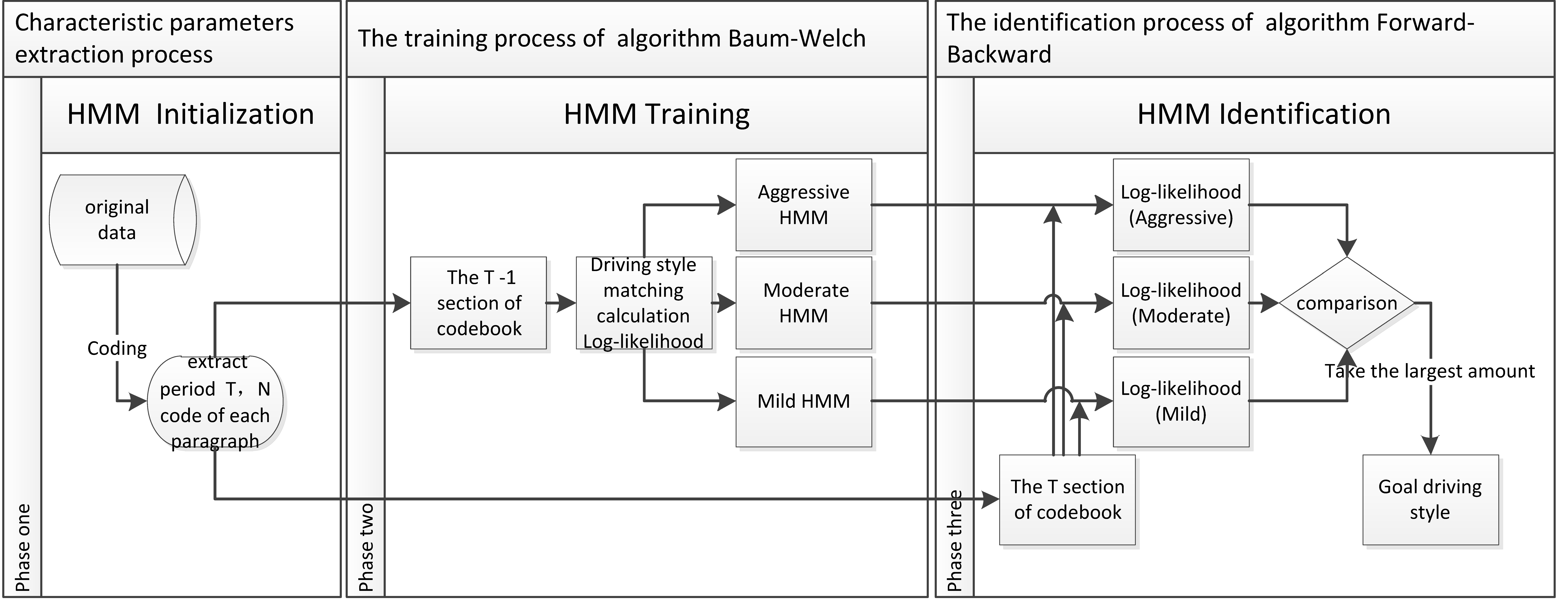

Supplement: S4 Fig — The one with the maximum probability is the corresponding driving style of this observation series. (TIF) [file pone.0182419.s004.tif]
