# Supplementary material for: Driving style recognition method using braking characteristics based on hidden Markov model
Source: PLoS One. 2017 Aug 24;12(8):e0182419. doi: 10.1371/journal.pone.0182419 (PMC5570378; doi:10.1371/journal.pone.0182419)
Supplement: S1 Table — Table notes subjects’ driving style. (DOCX) [file pone.0182419.s008.docx]

| **S1 Table. Driving style of experimental subjects.** | | | |
| --- | --- | --- | --- |
| **Subjects** | **Driving style** | **Subjects** | **Driving style** |
| D01 | Mild | D16 | Mild |
| D02 | Mild | D17 | Moderate |
| D03 | Aggressive | D18 | Moderate |
| D04 | Moderate | D19 | Aggressive |
| D05 | Moderate | D20 | Aggressive |
| D06 | Mild | D21 | Moderate |
| D07 | Aggressive | D22 | Moderate |
| D08 | Aggressive | D23 | Aggressive |
| D09 | Mild | D24 | Moderate |
| D10 | Aggressive | D25 | Aggressive |
| D11 | Mild | D26 | Aggressive |
| D12 | Moderate | D27 | Mild |
| D13 | Mild | D28 | Moderate |
| D14 | Moderate | D29 | Aggressive |
| D15 | Mild | D30 | Mild |
| Table notes subjects' driving style. | | | |
