# Supplementary material for: Driving style recognition method using braking characteristics based on hidden Markov model
Source: PLoS One. 2017 Aug 24;12(8):e0182419. doi: 10.1371/journal.pone.0182419 (PMC5570378; doi:10.1371/journal.pone.0182419)
Supplement: S2 Table — Accuracy μ=Taggressive+Tmoderate+TmildS. (DOCX) [file pone.0182419.s009.docx]

| **S2 Table. Comparison of recognition accuracy (%).** | | | |
| --- | --- | --- | --- |
| **Experimental subjects** | **HMM** | **ANN** | **SVM** |
| Aggressive (10 drivers) | 83.32 | 74.99 | 83.32 |
| Moderate (10 drivers) | 99.90 | 81.85 | 81.84 |
| Mild (10 drivers) | 83.35 | 75.01 | 83.33 |
| Overall average (30 drivers) | 88.86 | 77.27 | 82.83 |
| Accuracy $\mu=\frac{T_{aggressive}+T_{moderate}+T_{mild}}{S}$. | | | |
