# Supplementary material for: Driving style recognition method using braking characteristics based on hidden Markov model
Source: PLoS One. 2017 Aug 24;12(8):e0182419. doi: 10.1371/journal.pone.0182419 (PMC5570378; doi:10.1371/journal.pone.0182419)
Supplement: S3 Table — Calculated by the MATLAB command tic/toc recognition program. (DOCX) [file pone.0182419.s010.docx]

| **S3 Table. Comparison of recognition duration (s).** | | | |
| --- | --- | --- | --- |
| **Experimental subjects** | **HMM** | **ANN** | **SVM** |
| Aggressive (10 drivers) | 0.02 | 1.30 | 0.36 |
| Moderate (10 drivers) | 0.02 | 1.05 | 0.37 |
| Mild (10 drivers) | 0.02 | 1.40 | 0.32 |
| Overall average (30 drivers) | 0.02 | 1.25 | 0.35 |
| Calculated by the MATLAB command tic/toc recognition program. | | | |
